# Production and perception of legato, portato, and staccato articulation in saxophone playing

**DOI:** 10.3389/fpsyg.2014.00690

**Published:** 2014-07-15

**Authors:** Alex Hofmann, Werner Goebl

**Affiliations:** Institute of Music Acoustics (IWK), University of Music and Performing Arts ViennaVienna, Austria

**Keywords:** saxophone, articulation, music performance, timing, sensors, acoustics

## Abstract

This paper investigates the production and perception of different articulation techniques on the saxophone. In a production experiment, two melodies were recorded that required different effectors to play the tones (tongue-only actions, finger-only actions, combined tongue and finger actions) at three different tempi. A sensor saxophone reed was developed to monitor tongue-reed interactions during performance. In the slow tempo condition, combined tongue-finger actions showed improved timing, compared to the timing of the tongue alone. This observation supports the multiple timer hypothesis where the tongue's timekeeper benefits from a coupling to the timekeeper of the fingers. In the fast tempo condition, finger-only actions were less precise than tongue-only actions and timing precision of combined tongue-finger actions showed the higher timing variability, close to the level of finger-only actions. This suggests that the finger actions have a dominant influence on the overall timing of saxophone performance. In a listening experiment we investigated whether motor expertise in music performance influences the perception of articulation techniques in saxophone performance. Participants with different backgrounds in music making (saxophonists, musicians not playing the saxophone, and non-musicians) attended an AB-X listening test. They had to discriminate between saxophone phrases played with different articulation techniques (legato, portato, staccato). Participants across all three groups discriminated the sound of staccato articulation well from the sound of portato articulation and legato articulation. Errors occurred across all groups of listeners when legato articulation (no tonguing) and portato articulation (soft tonguing) had to be discriminated. Saxophonists' results were superior compared to the results of the other two groups, suggesting that expertise in saxophone playing facilitated the discrimination task.

## 1. Introduction

Producing expressive sound on single-reed woodwind instruments is a highly sophisticated motor task, requiring coordination between the fingers, aural cavity, and respiration (Scavone et al., 2008; Chen et al., 2011; Almeida et al., 2013). On the saxophone, a single reed of cane (or synthetic material), thinned on one end, is attached to the bottom side of a beak-shaped mouthpiece (Nederveen, 1998; Pinard et al., 2003). The player encloses the tip of the mouthpiece with his lips and blows into the tip opening. During sound production, the player's air stream excites the reed so that it oscillates related to the frequency of the impedance peak inside the instrument body (Fletcher, 1979; Dalmont et al., 2003; Almeida et al., 2010).

To perform expressively on woodwind instruments, the player may use a range of parameters to shape longer sequences of tones such as onset timing and tempo or the loudness of individual tones. An important dimension in woodwind performance is tongued articulation, thus referring to the way the tongue controls the shape of tone onsets, tone offsets, and the connections between tones (see Krautgartner, 1982; Bengtsson and Gabrielsson, 1983; Liebman, 2006). Goolsby (1997) reported that more than 21% of professional band rehearsal time is spend on instructions of articulation.

Articulation techniques on saxophone can be grouped in two main types: tongued articulation techniques and articulation without tonguing. *Legato* articulation does not involve tonguing and its sounding result is the smoothest note transition. Herby, only changes of the fingerings determine the timing and precision of note transitions. Professional fingering technique is required to produce smooth and clean *legato* tone-transitions on wind instruments (Almeida et al., 2009). For tongued note transitions, the intensity and duration of the tongue stroke defines the sounding result. *Portato* articulation is produced by soft tongue strokes to the vibrating reed, while the player blows constantly. The sound of consecutive *portato* tones has been described to be close to that of legato; the subdivision of tones is very subtle. Liebman (2006) gives instructions to the technique of tonguing on saxophone as follows: “it is the front portion of the tongue containing muscle tissue which flaps upward stroking the reed.” The resulting effect is that “the reed's motion and sound are momentarily stopped. The actual sounding of the articulation comes with the release of the reed” (p. 28). In contrast to the soft sound of portato separated tones, *staccato* tones are sharp and short. These are produced by placing the tongue immediately back on the reed after the initial articulation. A consequence of these two different techniques of articulation (tongued, non-tongued), the timing of the performance is controlled either by tonguing or by the fingers.

Timing precision in the execution of complex movement patterns is essential for musicians to produce rhythm in a sequences of tones. Palmer et al. (2009) investigated the influence of finger trajectories on temporal accuracy in clarinet performance, but restricted their focus on *legato* articulation. They reported a positive relationship between peak accelerations of finger movements and temporal accuracy of the performance, and concluded that tactile information available to the fingers supports timing control, similar to observations made with piano players (Goebl and Palmer, 2008). In woodwind performance, the fingers have to be coordinated with tongue movements to produce expressive sound. Studies based on isochronous tapping tasks showed that a coupling of synchronous movements operated by multiple effectors improved temporal stability. Experiments by Ivry et al. (2002) showed that synchronous tapping with both hands improved temporal stability, compared to tapping with only one hand. Additional foot tapping enabled further temporal improvements. In the case of saxophone performance, when tongue and finger actions have to be coordinated, the multiple effector advantage may also be the case. In line with these findings, we hypothesize that there is a positive influence of combined tongue-finger actions on the temporal stability in saxophone performance. In this study we will investigate how different articulation techniques in single-reed woodwind performance affect performance timing.

Perception and action in human motor control are strongly connected. The motor theory of speech perception argues that human understanding of speech-based auditory stimuli is based on the ability to recognize related vocal tract movements required to produce equivalent sounds (Liberman and Mattingly, 1985; Galantucci et al., 2006). Neuroimaging studies have shown that brain areas active in speech production are also active for speech listening (Fitch et al., 1997; Fadiga et al., 2002; D'Ausilio et al., 2009). Similar observations have been made for the production and perception of music (see Manto et al. 2012 for an overview). Overlap in the neural regions active when professional pianists listen to familiar pieces and the regions active when they perform these pieces has been observed (Haueisen and Knösche, 2001). The link between production and perception of music has been further discussed in the theory of auditory-motor interaction for music making (Zatorre et al., 2007). Recent research has shown that musicians are superior in judging asynchrony between sound and body movements for performances on the instruments they master than for performances on other instruments (Bishop and Goebl, under review). Taking into account that professional saxophone players practice over a decade to acquire the skill level to produce fast tone sequences with fluent articulation, we hypothesize that this motor expertise may also improve the ability to perceive articulation in saxophone performance.

In this paper, we investigate articulation techniques in saxophone performances in two experiments. In a production experiment, we examine timing measures in relation to effector combinations (tongue, finger, and both), finger movement directions (pressing for tone onsets vs. releasing for tone onsets), and different articulation techniques (legato, portato, staccato). In a second experiment, we test whether motor expertise in a particular field (i.e., saxophone performance) influences the perception of different articulation techniques in recorded saxophone sounds.

## 2. Experiment 1: production task

### 2.1. Methods

#### 2.1.1. Participants

Seven female and twelve male graduate saxophone students from the University of Music and Performing Arts Vienna (*N* = 19, mean age = 23 years, range = 18–33 years) participated in this study. On average, the participants played their instrument for 10.7 years (range = 4.5–20 years) and practiced 1.9 h per day (*SD* = 0.97). Eleven saxophonists reported they play classical music only, while the remaining eight participants perform usually as members of jazz ensembles.

#### 2.1.2. Experimental design

**Figure 1:**
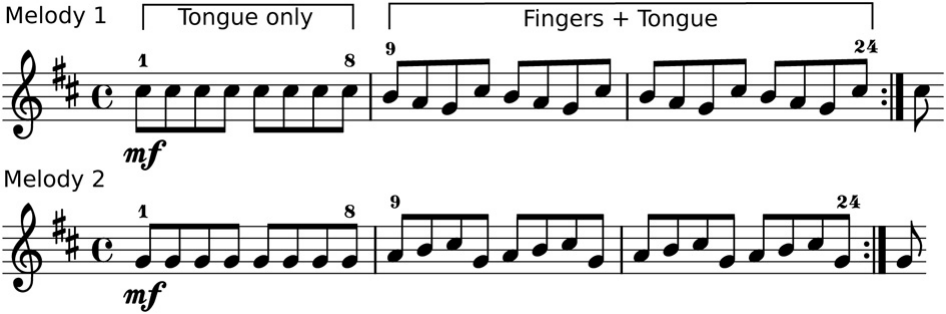
**Stimuli used for the production experiment**. Two 24-tone melodies in E-flat notation. Note numbers 1–8 require tonguing only. In melody one **(top)**, note numbers 9–24 require sequential key-depression by left-hand fingers. In melody two **(bottom)** a sequential finger lifting is required to open the tone-holes of the instrument.

Two isochronous 24-tone melodies were designed for the experiment. Both melodies consisted of the same elements (Figure 1): the first part (note number 1–8) is a tone repetition, produced by only tongue actions with no change of fingerings. The following notes (9–24) require a sequential depression (melody 1) or release (melody 2) of keys by left-hand fingers. Both melodies were given as a score for alto-saxophone (sounding a major sixth lower than notated), with additional portato, staccato, and legato articulation instructions. In legato articulation tone repetitions are not possible to play, thus note numbers 1–8 were omitted in the score.

#### 2.1.3. Equipment

**Figure 2:**
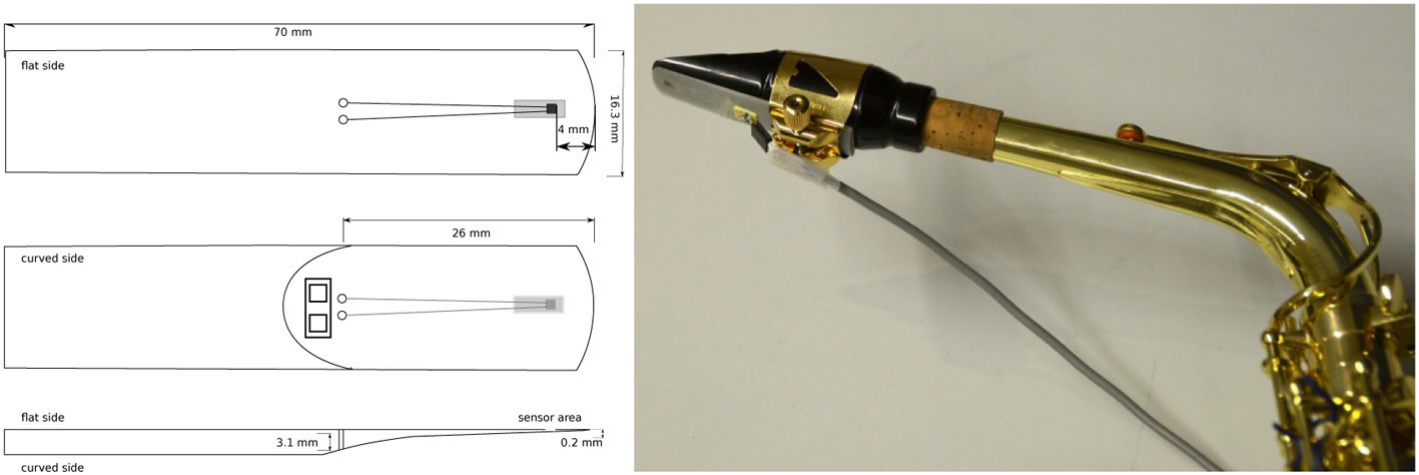
**Left:** Synthetic alto saxophone reed, equipped with strain gauge sensor (2 mm), glued with 4 mm distance from the tip on the flat side of the reed, to avoid direct lip/tongue contact with the sensor. **Right:** Mouthpiece with sensor reed used in the experiments to capture reed bending during performance.

The experimental set up consisted of a sensor-equipped alto-saxophone, a microphone, a digital metronome, and a multi-channel recording device. Strain gauge sensors (2 mm, 120 Ohms) attached to synthetic saxophone reeds (by Légère Reeds, Ltd.) were used to capture the bending of the reed during performance (Figure 2). The strain gauge was part of a Wheatstone quarter bridge circuit with 5 V (DC) power supply (Hofmann et al., 2013a). The sensor reed, the microphone (C414, by AKG Acoustics) and the digital metronome (KDM-1, by Korg Inc.) were connected via BNC cables to a multichannel analog-digital converter (DAQ LabView 2011, by National Instruments Corp.) to capture the signals simultaneously. All signals were recorded onto computer hard disk (A/D conversion with sampling rate 11.025 kHz, 16 bit resolution).

#### 2.1.4. Procedure

The experiment was conducted in accordance with the Declaration of Helsinki: Participants gave written consent prior to the experiment, played under normal performance conditions, and received a nominal fee at the end of the experiment.

In the beginning of the experiment, each player had to choose a synthetic saxophone reed out of four different reed-strengths (Légère: 2.0, 2.25, 2.5, 2.75). All saxophonists were allowed to use their own mouthpiece but played on the same alto-saxophone (77-SA, by Stagg). The metronome provided the synchronization signal on each quarter-note beat. The introduced tempi were 120 beats per minute (slow, IOI for eighth notes = 250 ms), 168 bpm (medium, IOI = 178.6 ms), and 208 bpm (fast, IOI = 144.2 ms). All participants got a 5 min warm-up, to practice the melody with the metronome at a slow tempo. For the experiment, each participant played both melodies in legato, portato, and staccato articulation. They synchronized with the metronome for two repetitions and continued playing when the metronome was muted, until the melody had been played 6 times in total. We recorded two trials per tempo condition, ordered from the slowest to the fastest. The experiment lasted for approximately 1 h per participant. In total 4644 tones were recorded per player (2 melodies x 2 trials x 3 tempi x 3 articulations; containing 145 tones for portato and staccato and 97 tones for legato). After their performances, the participants filled in a questionnaire about their musical background and the experiences with the sensor saxophone.

#### 2.1.5. Data analysis

**Figure 3:**
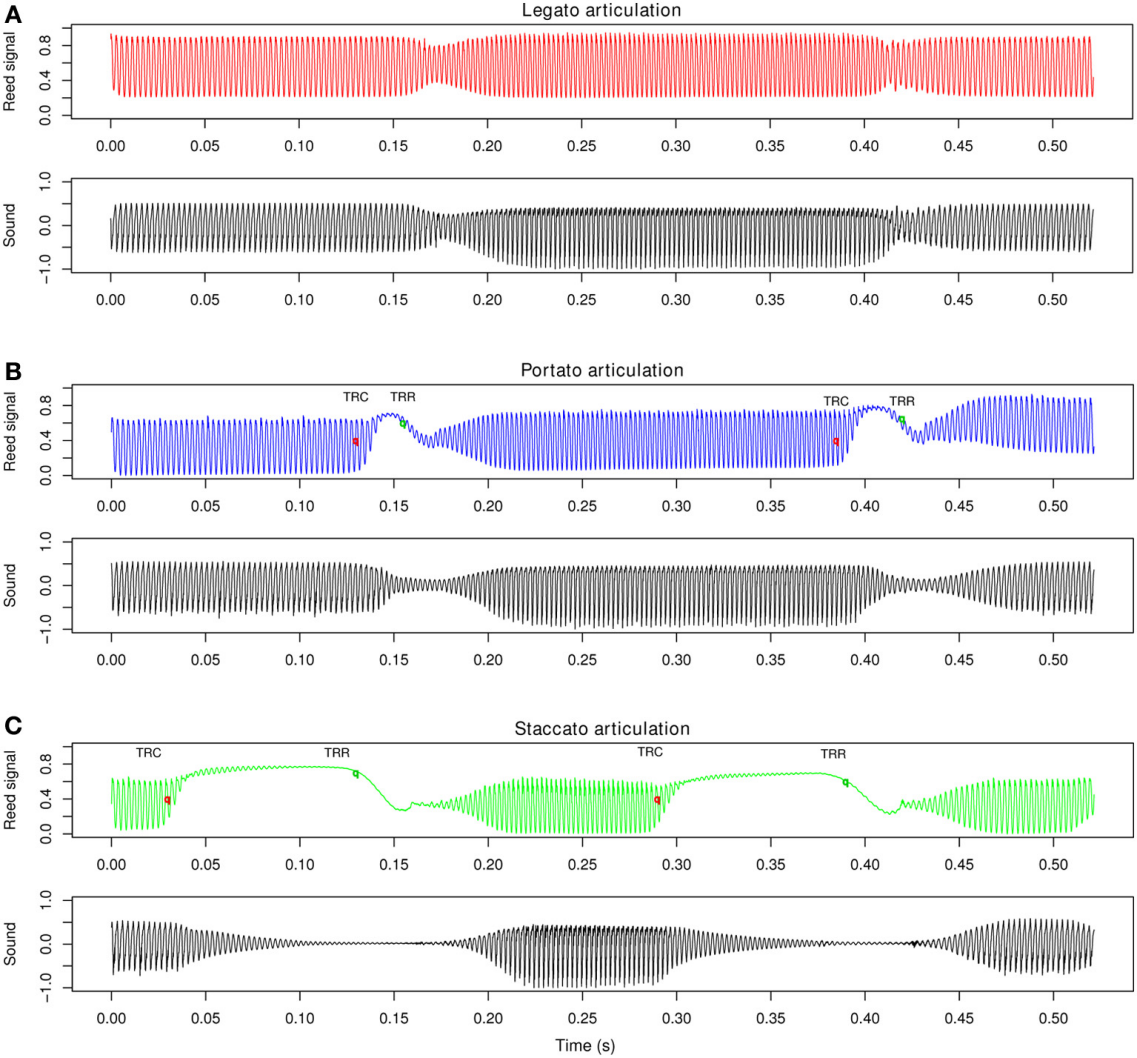
**Alto-saxophone sensor reed signals and radiated sound recorded in an anechoic chamber, showing a note transition (d2–e2–d2) under a tempo instruction of 250 ms inter-onset interval (audio sampling rate 44.1 kHz)**. Examples are taken from the pool of stimuli for the perception experiment in section 3. **(A)** Reed signal for legato articulation without tonguing (red) and radiated sound (black); **(B)** Reed signal (blue) for portato articulation with tongued note onsets (tongue reed release, TRR) and note offsets (tongue reed contact, TRC) and radiated sound (black); **(C)** Reed signal (green) for staccato articulation, with extended tongue-reed contact duration and radiated sound (black).

Sensor equipped saxophone reeds were used to capture the bending of the vibrating reed during human performance. Figure 3 shows typical sensor reed signals in relation to the radiated sound under three different articulation techniques. In legato articulation (Figure 3A) no tongue stroke was performed, contrary to portato articulation (Figure 3B), and staccato articulation (Figure 3C). The tongue strokes to the vibrating reed are visible in the captured signals, because the tongue presses the reed toward the mouthpiece lay and thereby damps the reed vibrations. We define two characteristic landmarks: first, a tongue-reed contact (TRC), when the tongue touches the reed, second, a tongue-reed release (TRR), when the tongue releases the reed and initiates the succeeding tone.

The data captured during the experiment contained more than 88,000 played tones, which makes a manual transcription impossible. A multiresolution analysis (MRA) based on wavelet methods has been used successfully for the analysis of various time critical signals, ranging from medical data (i.e., ECG time series, Percival and Walden, 2006) to transcriptions of drum patterns in audio recordings (Kronland-Martinet et al., 1987; Tzanetakis et al., 2001; Paradzinets et al., 2006). The following section discusses a landmark detection function (LDF) based on a wavelet decomposition of the sensor reed signal, where the external libraries (wmtsa, msProcess) were used in the R-statistics software package (R Core Team, 2013).

**Figure 4:**
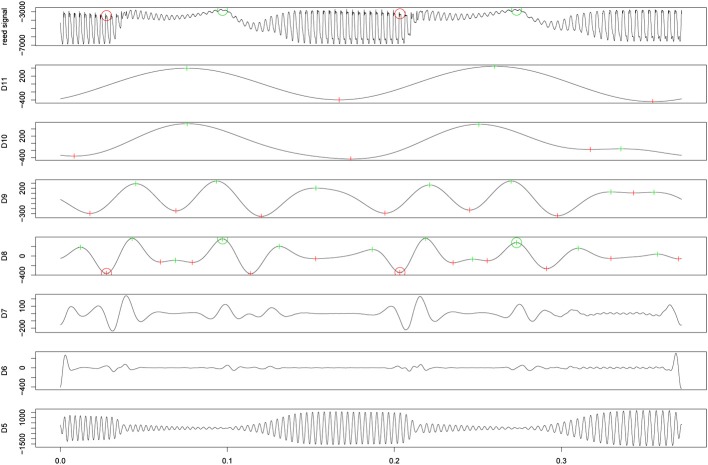
**Maximal Overlap Discret Wavelet Transform of a sensor reed signal containing tongued articulation: the figure shows the input signal (top) including detected landmarks (TRC: red circle, TRR: green circle) and the details of the wavelet decompositon $\tilde{D}$_11-5_ (below)**. The landmark detection function labeled maxima (green) and minima (red) in detail $\tilde{D}$_11_. These positions were refined to extrema of $\tilde{D}$_10_, $\tilde{D}$_9_, and $\tilde{D}$_8_.

The reed signal was decomposed using the Maximal Overlap Discrete Wavelet Transform (MODWT) of level *J*_0_ = 11. A Daubechies least asymmetric 8-tap filter LA(8) allows direct reference from the MODWT details to actual times in the reed signal. Figure 4 shows the algorithm of the LDF, working in two main steps. First, extrema in detail $\tilde{D}$_11_ (time resolution: △*t* = 92.88 ms) were labeled. These extrema represent reed displacements caused by the tongue. Hereby, maxima of $\tilde{D}$_11_ were labeled as TRR, because the following signal decrease is an indicator that the player released the tongue. Minima were labeled as TRC because a contact with the reed must have happened before releasing the reed. Second, landmarks were shifted to the extrema in details with a higher time resolution ($\tilde{D}$_10_, $\tilde{D}$_9_, and $\tilde{D}$_8_: τ_8_△*t* = 11.61 ms). A special treatment of the legato recordings was required to locate tone transitions without tongue actions. To ensure comparable detection results, the same MODWT analysis was applied to the legato recordings, but with an adapted LDF which worked on details $\tilde{D}$_10_, $\tilde{D}$_9_, and $\tilde{D}$_8_ only. One participant's data had to be omitted completely from the analysis, as the sensor data indicated that no tonguing was used in any of the playing conditions.

To evaluate the quality of the LDF, it was tested on a small data set which contained 2020 manually annotated landmarks. Starting from the annotated ground truth, the existence and number of detected landmarks around the annotated events was checked. The standard measures *precision*, *recall*, and *F-measure* were used. *Recall* describes the completeness of the search and *precision* gives status about the quality of the search results. *F-measure* combines the two previous measures. Overall, the wavelet-based analysis gave satisfactory results of the detection tasks with *F-measure* > 94% (see Hofmann et al. 2013b for details on the analysis of the sensor reed signals). To check possible influences of the LDF to the regularity of the extracted landmarks, we calculated the time differences of all detected landmarks to the manually annotated landmarks of the ground truth data set. A mean deviation of 0.42 ms (*SD* = 6.84) showed that the detected landmarks were close to the annotated landmarks.

### 2.2. Results and discussion

#### 2.2.1. Timing of performed melodies

To examine timing of the produced sequences, we calculated inter-onset intervals (IOI, in ms), as the time interval between two subsequent TRR (onset) landmarks (*IOI_x_* = *t*_*x* + 1_ − *t_x_*). From these IOIs we calculated the timing error (accuracy) and the coefficient of variation (CV, precision). The timing error (*IOI_obs_* − *IOI_exp_*)/*IOI_exp_* describes the relative deviation from the given tempo. A negative value corresponds to a sequence played too fast; a positive value to a sequence played too slow. The temporal precision of the played melody was calculated (*CV* = *SD_IOI_*/*Mean_IOI_*) to examine the regularity of the tone-events. *CV* values close to zero correspond to high regularity in the sequence, while larger values indicate higher variability in the onset distribution.

**Figure 5:**
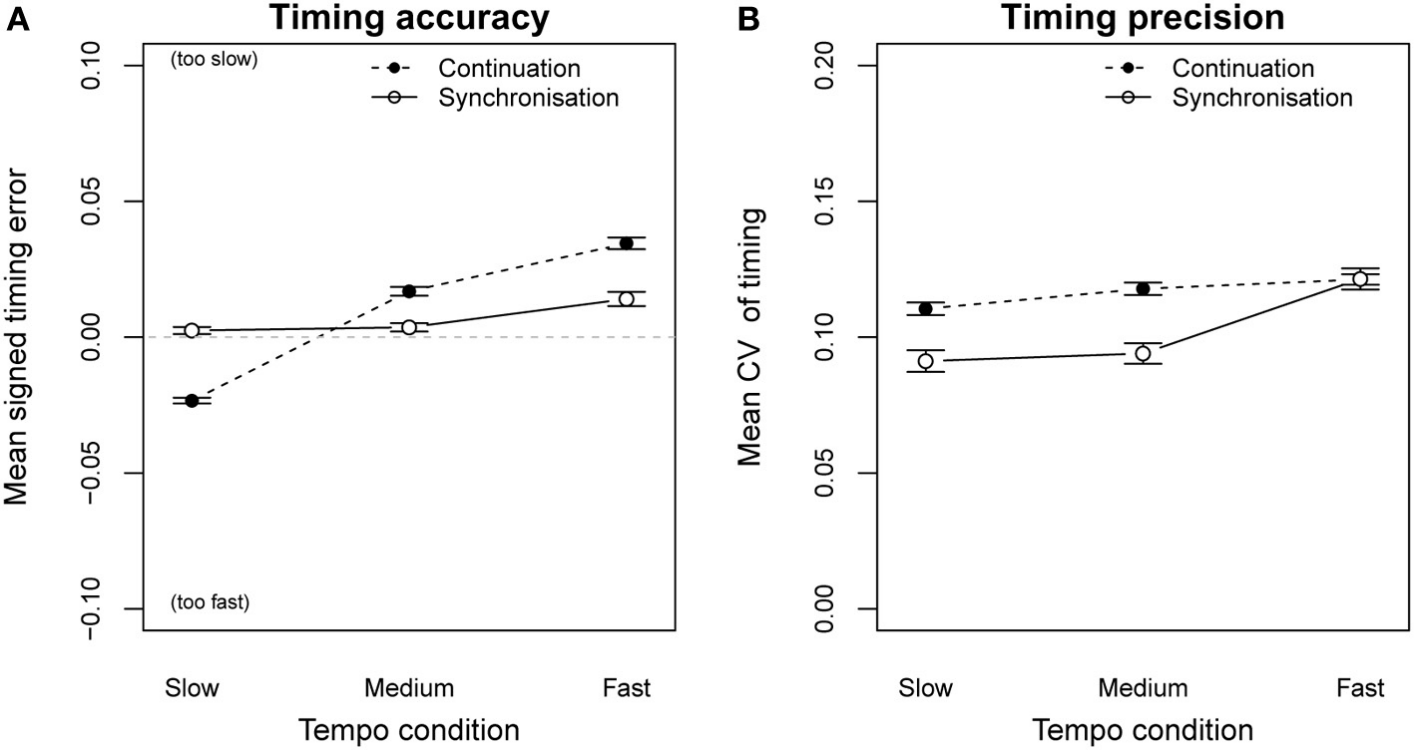
**Timing error (A) and coefficent of variation (B), for synchronization-continuation playing conditions**. When playing with metronome click (synchronization phase, solid line) and without metronome click (continuation phase, dashed line). Error bars show the standard error of the mean.

The average signed timing error of all performances during the synchronization phase was close to zero (*M* = 0.0077, *SD* = 0.032). Figure 5A (solid line) shows that all participants were able to play the melodies together with the metronome click in all three tempi. A Two-Way repeated measures analysis of variance (ANOVA) on timing error by tempo condition (metronome IOI = 250 ms, 178.6 ms, 144.2 ms) and synchronization condition (with metronome = synchronization, without metronome = continuation) indicated a significant main effect of tempo [*F*_(2, 34)_ = 13.23, *p* < 0.001] as well as a significant interaction between tempo and synchronization *F*_(2, 34)_ = 35.77, *p* < 0.001. Without metronome click, participants increased the playing speed in the slow tempo condition and reduced fast tempi to a more comfortable playing speed (Figure 5A, dashed line). Similar observations have been reported for performances on other instruments (e.g., piano performance, Goebl and Palmer 2013). The overall temporal precision of the played sequences *CV* = (*SD_IOI_*/*Mean_IOI_*) was high (mean *CV* = 0.11). The same Two-Way ANOVA was calculated for the CV and revealed a significant main effect of synchronization condition [*F*_(1, 17)_ = 19.61, *p* < 0.001] and a significant interaction between tempo and synchronization *F*_(2, 34)_ = 6.92, *p* < 0.01. Figure 5B (dashed line) shows the reduction of timing precision even for moderate playing speeds when the metronome click was removed.

#### 2.2.2. Timing with multiple effectors

The melodies (Figure 1) were designed to consist of three distinct parts which had to be played with the fingers only (legato), the tongue only (portato note repetition), and with tongue and fingers in a coordinated fashion (descending and ascending note sequence in portato articulation). For this analysis we restricted our data set to legato and portato recordings, because the onset detection for staccato melodies was less robust.

**Figure 6:**
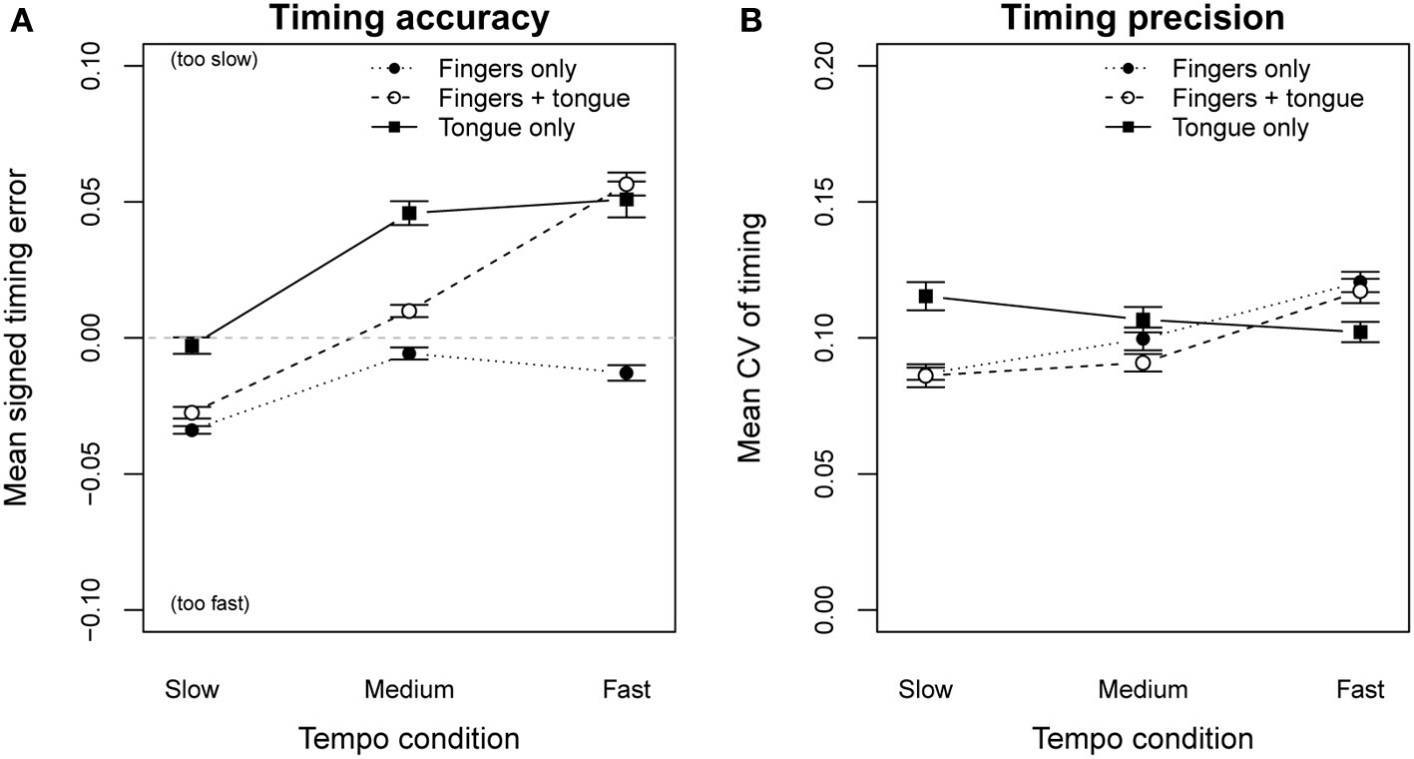
**Timing error (A) and coefficient of variation (B), grouped by effectors used to produce tone onsets**. Error bars show the standard error of the mean.

We grouped parts of the melodies according to the effectors required for playing and compared onset timing between these parts. A Two-Way repeated measures ANOVA on timing error by effector combination and tempo, indicated a significant main effect of the executing effector [*F*_(2, 34)_ = 25.05, *p* < 0.001], as well a significant interaction between effector and tempo [*F*_(4, 68)_ = 28.39, *p* < 0.001; Figure 6A]. A *post-hoc* pairwise *t*-test verified a significant influence of each effector condition on the timing error (Bonferoni: *p* < 0.001). Playing with only finger actions led to faster performances than the metronome in all three tempo conditions (mean timing error = −0.017). Tongue-only actions led to slower performances compared to the metronome (mean timing error = 0.026), especially in the medium and the fast playing conditions (*M* = 0.045). Using both effectors in a coordinated fashion (tongue + fingers) stabilized the timing error (*M* = 0.013), but showed a significant tempo reduction in the fast tempo condition (*M* = 0.058). We observed a learning effect in the recording of the second trial for the same task [*F*_(1, 17)_ = 6.55, *p* < 0.05; see Table 1]. The timing error for combined tongue-finger actions at medium and fast tempi was significantly reduced in the second trial. This indicates that professional players already improved their tongue-finger coordination after the first 6 repetitions and were able to perform the second trial with reduced timing error.

**Table 1:** **Timing error for both trials of sequences performed with different effectors in three tempo conditions**.

**Tempo (IOI)**	**Slow (245 ms)**		**Medium (178.6 ms)**		**Fast (144.2 ms)**	
	**Trial 1**	**Trial 2**	**Trial 1**	**Trial 2**	**Trial 1**	**Trial 2**
Fingers only	−0.03332	−0.03430	−0.00095	−0.01010	−0.01170	−0.01402
Fingers w. Tongue	−0.02675	−0.02760	**0.01610**	**0.00361**	**0.06659**	**0.05029**
Tongue only	−0.00666	−0.00521	0.04549	0.03438	0.04636	0.04446

*Bold numbers depict improved timing error (learning effect) for combined tongue-finger actions at medium and fast playing speeds for the second trial*.

A Two-Way repeated measures ANOVA on temporal precision (CV) by effector combination and tempo, showed a significant effect of tempo [*F*_(2, 34)_ = 5.76, *p* < 0.01], and an interaction between tempo and the used effectors [*F*_(4, 68)_ = 6.57, *p* < 0.001; Figure 6B]. Looking at the *CV* values plotted in Figure 6B (dotted line), we see that timing precision for the finger-only condition was lower in the slow tempo condition than in the fast tempo condition. A similar pattern appeared for the tongue-finger condition. Contrary, tones played only by tonguing (solid line) showed almost a constant irregularity over all three tempo conditions. This was confirmed by three separate One-Way repeated measures ANOVAs on the CV by tempo condition. The results showed a significant main effect of tempo for both conditions where fingers were involved [fingers only: *F*_(2, 34)_ = 13.25, *p* < 0.001; tongue + fingers: *F*_(2, 34)_ = 5.51, *p* < 0.001], but no significant effect of tempo under the condition of playing with the tongue alone. Separate *post-hoc* pairwise *t*-tests, three for each tempo condition, showed that at the slowest and fastest tempo the tongue-only condition was significantly different from the other two conditions (Bonferoni: *p* < 0.05), except from combined tongue-finger actions in fast tempo (*p* = 0.211). There was no significant difference between the finger-only condition and the combined tongue-finger condition across all tempi. These findings suggest that at slow tempi timing improves with combined tongue-finger actions. The observed effect can be explained by the multiple-timer model (Ivry et al., 2002), where the timer responsible for the tongue movements is coupled to the timer of the fingers. Such a coupling of multiple effectors has been shown to improve timing precision. We found the opposite effect for the fast tempo condition: Tongue timing deteriorated, when combined with finger movements. This fast tempo condition (IOI = 144 ms) examined performances close to the *synchronization threshold* of professional musicians (100–120 ms, Repp, 2005). The measured *CV* values were about the same level as tones played with only fingerings. It is interesting that professional saxophonists were able to produce coordinated movements under this extreme tempo condition, but did not benefit from the coupling of the tongue to the fingers. Hence, saxophonists' tongue movements were coupled to the finger movements, even if the precision of the finger movements was worse than the precision of the tongue alone. This indicates that in saxophone playing, the timing precision of the fingers dominates the precision of the overall performance, thus overruling the timing effects of the tongue.

#### 2.2.3. Timing and the direction of finger motion

To play descending tone sequences on the saxophone, keys have to be pressed, while ascending sequences require fingers to open tone holes. To see if the direction of finger movements (pressing down vs. lifting up) influences the timing of the performance, we contrasted (legato) sequences with a focus on key depression (Melody 1) to those focussed on lifting the keys (Melody 2). A Two-Way repeated measures ANOVA on timing error showed no significant effect of the direction of finger motion nor any interactions with tempo. The same ANOVA on timing precision showed no significant effects. Similar observations have been reported for clarinet performances by Palmer et al. (2009).

#### 2.2.4. Characteristics of articulation techniques

**Figure 7:**
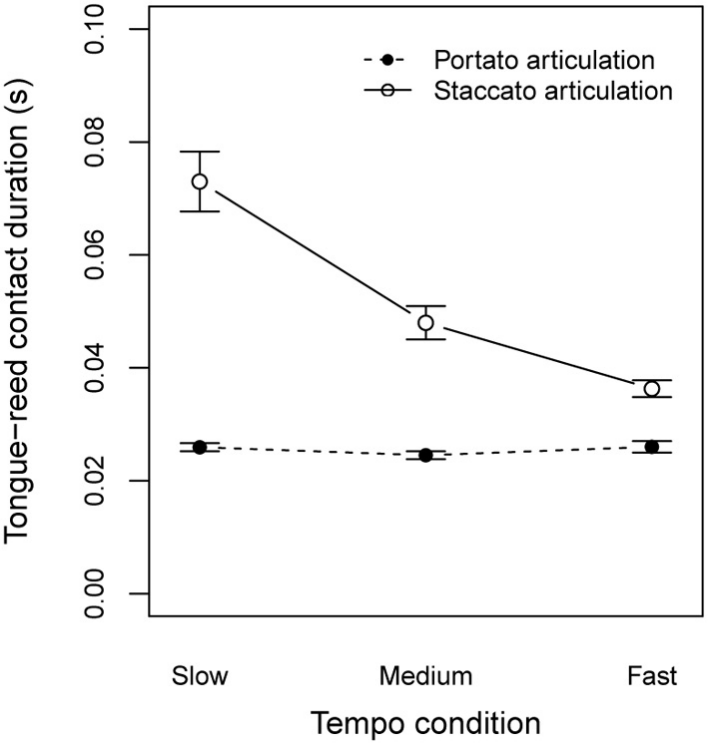
**Tongue-reed contact duration under different tempo conditions for portato articulation and staccato articulation**. Error bars show the standard error of the mean.

We recorded reed signals of melodies with legato, portato, and staccato articulation (Figure 3). Whereas for legato articulation, no tongue actions were required, portato and staccato note transitions required precise tonguing. Each of these articulation techniques allows variation within itself based on the onset and offset timing. Bengtsson and Gabrielsson (1983) discussed the resultant concepts of duration and emphasized the importance of controlling onset and offset parameters for the motion character of the rhythm. We investigated the tongue-reed contact duration (TRdur) for portato and staccato tone transitions by subtracting the TRC times (offset of previous tone) from TRR times (onset of subsequent tone, Figure 3). The average contact duration for portato articulation for all participants was 25.5 ms (*SD* = 4.1 ms, see Figure 7). A One-Way repeated measures ANOVA on TRdur by tempo condition showed no significant effect of tempo [*F*_(2, 34)_ = 1.3, *p* = 0.295]. On the contrary, the same ANOVA on staccato articulation showed a highly significant influence of tempo condition on the tongue-reed contact duration [*F*_(2, 34)_ = 25.2, *p* < 0.001]. In staccato articulation, the contact duration (gap between tones) varies with the tempo. We calculated the relative gap duration for each note transition by *TRdur*/*IOI_exp_* and calculated a One-Way repeated measures ANOVA on relative gap duration by tempo condition. The results showed no effect of tempo [*F*_(2, 34)_ = 1.7, *p* = 0.198]. The relative gap duration was in the range of 25–29% for all three tempo conditions (slow tempo: 0.29; medium tempo: 0.27; fast tempo: 0.25). This suggests that in portato articulation the tongue-reed contact duration remains constant, independent of the playing speed, while in staccato articulation, the relative gap duration is constant.

#### 2.2.5. Influences of the measurement setup to the performances

In the questionnaire, we asked how comfortable the participants felt while playing the sensor-equipped saxophone reed. The reed quality had to be rated between 1 (very good) and 7 (very bad). Results showed that the reed quality was evaluated as medium quality (*M* = 3, *SD* = 1.5). Participants also had to indicate whether they felt comfortable when playing the sensor instrument or not. We tested timing accuracy and timing precision for this group effect by separate between-subjects ANOVAs. We found no significant effect between the two groups, thus, the sensor instrument did not affect the recorded performances. We also tested for effects of self-reported handedness and skill level (years of playing the instrument), but found no significant effects.

## 3. Experiment 2: listening test

We were interested in the abilities of listeners with different expertise in music performance to discriminate between common articulation techniques in the sound of the saxophone. Furthermore, we were interested whether motor expertise in saxophone performance would facilitate the perception of saxophone articulation.

### 3.1. Methods

#### 3.1.1. Participants

Nineteen female and twelve male (*N* = 31, mean age = 24 years, range = 19–32 years) students from Vienna music conservatories and Vienna universities participated in the listening study. The group consisted of 10 saxophone players, 10 musicians that play an instrument other than the saxophone and 11 non-musicians. The saxophone players had a mean of 10.5 years (range = 5–15 years, *SD* = 3.06) of experience in playing their instrument: eight of them also participated in the production experiment described above (section 2). The group of musicians, who did not play a wind instrument, had a mean of 15.3 years (range = 8–22 years, *SD* = 3.83) of experience in musical practice of various instruments. The group of non-musicians were students of other fields, but 8 subjects had musical training in their early childhood, with a mean of 4.5 years. Only one of the non-musicians had experience with playing a wind instrument (the recorder).

#### 3.1.2. Experimental design

In a 3 × 3 × 2 × 2 × 2 (3 articulations × 3 intervals × 2 registers × 2 players × 2 listening blocks) design, we tested which articulation techniques our participants were able to discriminate. We recorded note transitions of three different pitch intervals (major second, major sixth, major sixth including register change), with legato, portato, and staccato articulation, within two registers by two different players (one of them also participated in the production experiment, the other is the first author of this paper) on the same alto-saxophone (YAS 32, by Yamaha Corp.), using their mouthpieces (AL3, by Vandoren; Original 7^*^3, by Claude Lakey). Recordings were made in an anechoic chamber using a microphone (C414, by AKG Acoustics) and Labview hardware and software (DAQ LabView 2011, by National Instruments Corp.) for recording the stimuli (44.1 kHz sampling rate, 16 bit resolution). Both players used synthetic sensor equipped saxophone reeds (section 2.1.3), to ensure tongue-reed contact in the portato and the staccato playing conditions. During the recordings, both players heard a metronome click on headphones (108 bpm for larger intervals, 120 bpm for small intervals), to produce consistent timing in the stimuli. We recorded three eighth-notes (two note transitions, see Figure 3) for each audio file. The beginning and the ending of the audio file was edited with a volume fade-in and fade out, to limit the sound of articulatory actions to only the two note-transitions. In total, 36 different audio files were comprised in the pool of stimuli.

Stimuli were presented to the participants in the form of an ABX listening test on a laptop computer. A java-based software program enabled the participants to click on one of 3 buttons (A-Button, B-Button, X-Button) to play back one stimulus. Buttons A and B contained two note transitions played with different articulation techniques. Button X contained a third recording that matched the articulation used in either A or B. The question our participants had to answer was: “Does X sound like A or B ?” Listeners had to decide whether X was more similar to A or B. Responses and reaction time were recorded by the software.

#### 3.1.3. Procedure

The experimental procedure complied with the Declaration of Helsinki: Participants gave written consent prior to the experiment. All participants worked on the same laptop computer (by ASUSTeK Computer Inc.) in a quiet environment and used the same studio headphones (K121, by AKG Acoustics). The could adjust the playback volume to a comfortable level. Each participant had 5 practice trials to learn how to navigate the ABX listening test software. A pop-up on the screen indicated when the actual experiment began. The experiment was grouped into two blocks, each containing all 36 stimuli in four different listening orders. Participants were allowed to play back the stimuli as often as required to make an assured judgment. After the first block was done the participants filled in a questionnaire about their musical background. Afterwards the participants made another set of judgements. The entire experiment lasted for about 30 min per participant.

### 3.2. Results and discussion

Overall, participants from all three groups were able to accomplish the listening test with over 87% of correct answers. A Chi-squared test revealed no significant difference on correct answers between the two repeated listening blocks [χ^2^_(1)_ = 2.59, *p* = 0.11]. No effects of listening order [χ^2^_(3)_ = 5.01, *p* = 0.17] or recording saxophonist [χ^2^_(1)_ = 0.05, *p* = 0.82] were found either. Due to a labeling mistake in the playback list of stimuli, one stimulus pair had to be excluded from the results^1^.

**Figure 8:**
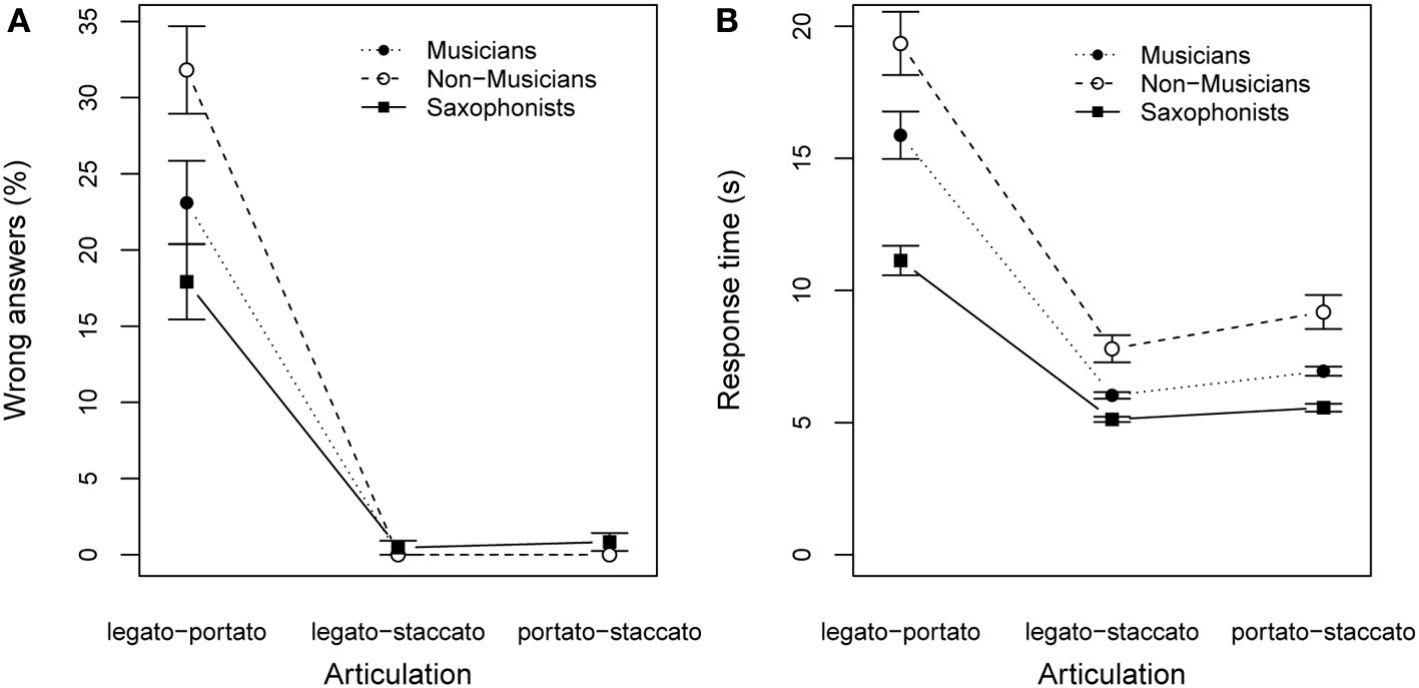
**Results of listening experiment: (A) percentage of mistakes for discrimination task; (B) response time to accomplish the task; Participants were grouped by their expertise in music making**. Error bars show the standard error of the mean.

To convert dichotomous response data to an interval-scale level, we computed the percentage of wrong answers per participant collapsing across listening blocks and players. A Two-Way ANOVA on percentage of wrong answers, with articulation (type of articulation to discriminate) as within-subjects and listeners expertise as between-subjects revealed a significant effect of the articulation [*F*_(2, 56)_ = 187.825, *p* < 0.001] and a significant effect of the listeners expertise [*F*_(2, 28)_ = 4.167, *p* < 0.05], as well as a significant interaction [*F*_(4, 56)_ = 5.847, *p* < 0.001; see Figure 8A]. A Two-Way ANOVA on the response duration with articulation as within-subjects and listeners expertise as between-subjects factor revealed a significant effect of articulation [*F*_(2, 56)_ = 67.897, *p* < 0.001], but no significant effect of expertise or interactions (see Figure 8B).

Focussing on articulation, *post-hoc* pairwise *t*-tests showed that results from the legato-portato listening task differed significantly from the results of the other two tasks (*p* < 0.001). Errors occurred most often when participants had to discriminate between legato articulation and portato articulation (25% wrong answers, compared to <1% wrong answers for remaining articulation types). Additionally, Figure 8B shows the highest response durations for the legato-portato condition.

Concering the listeners expertise, Figure 8 shows that non-musicians gave more wrong answers (32%) and required more time than the other two groups to respond (duration to answer per question *M* = 19.35 s, *SD* = 19.5), followed by non-wind-instrument players (23% wrong answers; *M* = 15.79 s, *SD* = 13.8) and saxophonists (18% wrong answers; *M* = 11.13 s, *SD* = 8.7). The results from our listening test suggest that musical expertise alters the ability to discriminate subtle sound differences, like between legato and portato tone transitions. The distinct sound of staccato tone transitions was well discriminated from the other two articulation techniques, by all three groups of listeners.

## 4. General discussion

This study investigated the production and the perception of articulation on the saxophone with two experiments. For the production experiment we built a sensor equipped saxophone reed to monitor tongue-reed interaction in alto-saxophone performance, while participants performed melodies at three tempi with different articulation techniques. The captured sensor-reed signals showed that for portato articulation, the tongue-reed contact duration was independent from the given tempo, whereas for staccato articulation the gap between the tones was relative to the given tempo. In legato articulation, no tongue stroke occurred, and tone transitions were initiated by a change of the fingerings. Such coordination tasks occur with all wind instruments, where different effectors (tongue and fingers) are required to produce one tone (e.g., flute, clarinet, trumpet). It is also the case for string instruments that the player has to coordinate different effectors to produce one tone. Bowing movements with the right arm have to be coordinated with left hand fingerings. Baader et al. (2005) looked at bow-finger coordination in violin playing and recorded tone sequences where subjects had to play a sequence of tones, in which each tone was initiated with a bow stroke and a finger change. The focus of their study was primarily on bow-finger synchronization, which was shown to be far from perfect simultaneity (50 ms), but did not lead to audible interruptions. To play larger intervals on woodwind instruments the player has to close or open multiple tone holes at the same time. This requires simultaneous finger movements, also called *safe finger transitions*. Almeida et al. (2009) showed that for flute performance *unsafe finger transitions* with approximately 21 ms already lead to audible changes in the radiated sound. Taking these studies into account, it seems that wind instrumentalists need even more precise finger movements, which additionally have to be coordinated with the movements of tongue.

In our study we looked into temporal effects of saxophone performances under different tempi, which were produced by different effectors (fingers only, tongue only, combined tongue-finger actions). We found that at the slow tempo, tone onsets produced by tongue-only actions were significantly less precise than tone onsets produced by fingerings only. Highest precision was archived for combined tongue-finger actions. This corresponds to our hypothesis that timing precision improves for combined tongue-finger actions. However, we did not expect to see that in the fast tempo condition, tonguing alone was more precise than finger-only actions and combined tongue-finger actions showed a high timing variability, at approximately the same level as finger-only actions. This finding suggests that fingers play a dominant role in the overall timing of saxophone performances.

In woodwind performance finger actions usually do not receive the same attention as with piano playing, where the finger movements directly produce the sound. Our observation that there is a strong influence of finger timing on the overall timing in woodwind performance may put a new focus on further investigations of finger movements, finger trajectories, and finger forces in this domain. With the help of sensor-equipped wind instruments and the development of new customized sensors, useful advice for music education may be gained in future research.

In the listening experiment, we observed that the articulatory sound modifications (legato, portato, staccato) were mostly perceivable for non-musicians, musicians (not playing saxophone) and professional saxophonists. Only the sound of portato tongue-reed strokes was difficult to discriminate from that of non-tongued legato tone-transitions. There are two possible reasons for this. First, a brief damping of the reed vibrations does not immediately stop the standing wave in the resonator and thus only slightly modifies the radiated sound. Not all listeners notice that the reed has been stopped. Second, *unsafe finger transitions* in legato playing may also cause small gaps in the sound, which non-experts may confound with portato tonguing (Almeida et al., 2009). Nevertheless, the group of professional saxophonists was superior in discriminating legato from portato sounds. An interesting observation was that the one participant, who did not use any tonguing during the production experiment and was therefore excluded from analysis there, also showed the worst results in the listening experiment (in the group of professional saxophone players). This strengthens our assumption that expertise in the underlying motor-actions to modify sound, facilitates perceptional discrimination of such sound modifications. This conclusion is in line with the motor theory of speech perception (Galantucci et al., 2006): the link between perception and production of speech may also apply for the perception of articulation in saxophone music performance. As a consequence, learning to play a musical instrument enhances the ability to perceive more details of musical performance on that instrument.

## Funding

This research was supported by the Austrian Science Fund (FWF): P23248-N24.

### Conflict of interest statement

The authors declare that the research was conducted in the absence of any commercial or financial relationships that could be construed as a potential conflict of interest.

## Footnotes

^1^A, B, and X stimuli contained three different articulations: no correct answer was possible.
